# Inhibition of SYK kinase does not confer a pro-proliferative or pro-invasive phenotype in breast epithelium or breast cancer cells

**DOI:** 10.18632/oncotarget.27545

**Published:** 2020-04-07

**Authors:** David J. Lamb, Aleksander Rust, Albin Rudisch, Tobias Glüxam, Nathalie Harrer, Herwig Machat, Ingrid Christ, Florian Colbatzky, Andreas Wernitznig, Annika Osswald, Wolfgang Sommergruber

**Affiliations:** ^1^Immunology & Respiratory, Boehringer Ingelheim Pharma GmbH & Co. KG, 88397 Biberach an der Riß, Germany; ^2^Cancer Biology and Genetics Program, Memorial Sloan Kettering Cancer Center, New York, NY 10065, USA; ^3^Cancer Cell Signalling, Boehringer Ingelheim RCV GmbH & Co KG, A-1121 Vienna, Austria; ^4^Department of Medicine I, Comprehensive Cancer Center, Medical University of Vienna, 1090 Wien, Austria; ^5^Non-clinical drug safety, Boehringer Ingelheim Pharma GmbH & Co. KG, 88397 Biberach an der Riß, Germany; ^6^Translational Medicine and Clinical Pharmacology, Boehringer Ingelheim Pharma GmbH & Co. KG, 88397 Biberach an der Riß, Germany; ^7^Biotechnology, University of Applied Sciences, 1030 Vienna, Austria

**Keywords:** SYK inhibitor, breast carcinoma, 3D culture, murine mammary gland, proliferation

## Abstract

SYK has been reported to possess both tumour promotor and repressor activities and deletion has been linked to a pro-proliferative / pro-invasive phenotype in breast tumours. It is unclear whether this is a consequence of protein deletion or loss of kinase activity. The SYK inhibitor, BI 1002494, caused no increase in proliferation in breast cancer cells or primary mammary epithelial cells in 2D or 3D cultures, nor changes in proliferation (CD1/2, CDK4, PCNA, Ki67) or invadopodia markers (MMP14, PARP, phospho-vimentin Ser56). BI 1002494 did not alter SYK protein expression. There was no change in phenotype observed in 3D cultures after addition of BI 1002494. Thirteen weeks of treatment with BI 1002494 resulted in no ductal branching or cellular proliferation in the mammary glands of mice. An *in silico* genetic analysis in breast tumour samples revealed no evidence that SYK has a typical tumour suppressor gene profile such as focal deletion, inactivating mutations or lower expression levels. Furthermore, SYK mutations were not associated with reduction in survival and disease-free period in breast cancer patients. In conclusion, small molecule inhibition of the kinase function of SYK does not contribute to a typical tumour suppressor profile.

## INTRODUCTION

Spleen tyrosine kinase (SYK) is a member of the Syk family of non-receptor cytoplasmic tyrosine kinases [1], primarily expressed in hematopoietic tissues but also in a variety of different tissues [2] including normal epithelial breast tissue [3]. SYK propagates signal transduction for a number of immunoreceptor tyrosine-based activation motif (ITAM)-dependent pro-inflammatory pathways, including Fc receptor, B-cell receptor and integrin signalling [1]. A novel role for SYK has also recently been reported in the signal transduction for T-cell receptors [4]. Interestingly, the expression level of SYK was found to be significantly higher in untreated healthy lymphocytes of BRCA1 heterozygote carriers compared with controls [5]. SYK, therefore, was suggested as a candidate for better diagnosis and treatment of BRCA1 mutation-associated breast cancer.

SYK has been proposed to possess both a tumour suppressor and tumour promotor activities, depending upon the type of malignancy (reviewed in [6]). On one side, B-cell receptor signalling has been associated with survival signals in malignancies of B cell origins, and as a consequence a number of SYK inhibitors have entered clinical development for the treatment of B-cell malignancies, including entospletinib [7] and TAK-659, although very recent data from a Phase II study in refractory diffuse large B-cell lymphoma suggests that efficacy of the relatively unselective SYK inhibitor, fostamatinib, was poor [8]. In contrast, a number of studies have suggested a negative regulatory role of SYK in the control of cell proliferation, migration and invasion in breast cancer cell lines [6, 9–11]. Allelic deletion of SYK has been reported to be associated with higher incidences of breast cancer [12]. It has been further reported that loss of SYK expression through promoter hyper-methylation is associated with increased invasiveness of breast cancer cells [11, 13, 14]. However, the case for SYK possessing tumour suppressor activity relies on data in which SYK (or SYK-containing allele) is deleted and/or silenced by e. g. hypermethylation. It is therefore unclear whether the putative tumour suppressor phenotype associated with SYK deletion is conferred via loss of kinase activity or of the protein itself.

The proximal location of SYK in these multiple immune pathways confers a broad range of pro-inflammatory activity and makes inhibition of SYK kinase activity an attractive therapeutic target for a range of inflammatory diseases such as rheumatoid arthritis and asthma [15–18] but also for some haematological tumours such as non-Hodgkin lymphoma [19, 20]. Given the requirement for chronic dosing regimens to control inflammatory diseases, it is important to understand whether any putative tumour suppressor function relies on the presence of the protein *per se*, or on its kinase function.

In these studies, we sought to address this question by two means: (1) by assessing the effect of a SYK inhibitor (BI 1002494) on proliferation and invasiveness markers in normal mammary epithelial cells and a number of breast cancer cell lines including long-term treatment of naïve adult mice, and (2) reviewing the Cancer Genome Atlas (TCGA) [21] database to compare genetic characteristics of SYK and the well-known tumour suppressors TP53 and PTEN in samples from invasive breast cancer.

BI 1002494 is a potent and selective inhibitor of SYK [22]. In a kinase inhibition assay the IC_50_ of BI 1002494 is1nM and in whole blood basophil and B-cell activation assays is 114 nM and 810 nM, respectively. At 1 μM, BI 1002494 inhibits just 22 kinases in the Invitrogen panel of 306 kinases by more than 50%, conferring a favourable potency and selectivity profile over other SYK inhibitors described in the literature [22].

## RESULTS

Experiments were performed with either immortalized or tumorigenic breast cell lines [MCF-10A (pre-neoplastic mammary epithelial cell line), DU4475 (highly invasive mammary gland carcinoma cell line derived from a skin metastatic site), MDA-MB-468 (moderately invasive breast adenocarcinoma cell line derived from a pleural metastatic site) and T-47D (invasive breast adenocarcinoma cell line derived from a pleural metastatic site)], or primary human mammary epithelial cells.

### Acute incubation of the non-tumorigenic epithelial cell line MCF-10A and breast cancer cell lines MDA-MB-468 and DU4475 with BI 1002494 does not lead to a dose-dependent increase in proliferative or EMT/invadopodia markers

**Figure 1:**
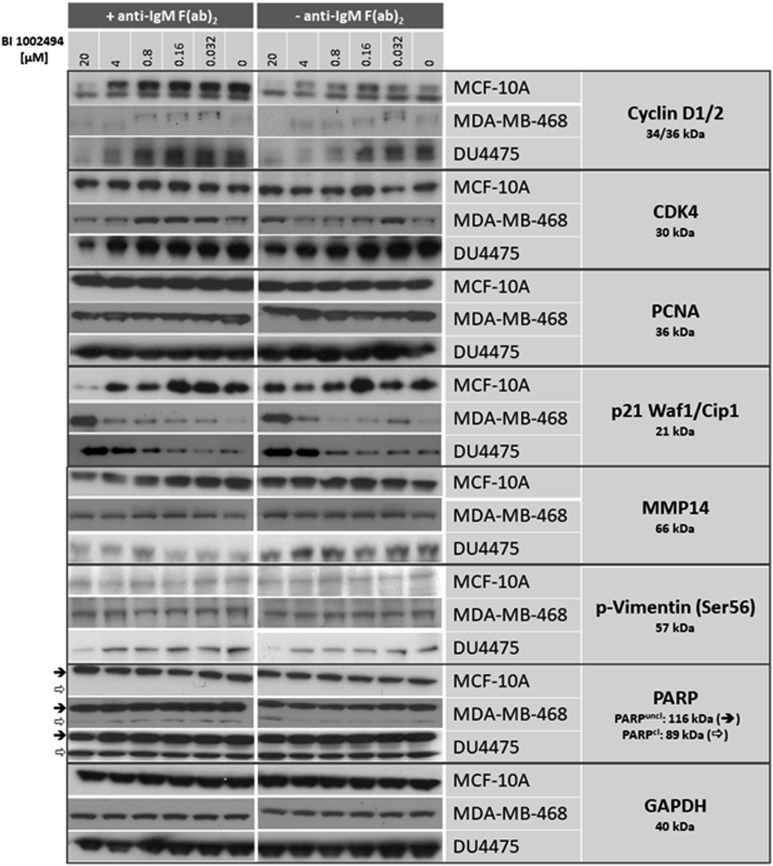
Western blot: effect of 16-hour incubation of BI 1002494 (0, 0.032, 0.16, 0.8, 4 and 20 μM) on selected proliferative (cyclin D1/2, CDK4, PCNA, p21 Waf1/Cip1) and EMT/invadopodia (MMP14, PARP, phospho-vimentin Ser56) marker proteins in a non-tumorigenic, spontaneously immortalized human breast epithelial cell line MCF-10A and in two breast cancer cell lines MDA-MB-468 and DU4475. + anti-IgM F (ab)_2_ indicates the presence of 10 μg/mL, -anti-IgM F (ab)_2_ its absence.

MCF-10A, MDA-MB-468 and DU4475 cells were incubated for 16 hours with 0–20 μM BI 1002494. In none of the cell lines was a dose-dependent increase observed for the expression of proliferation markers such as cyclin D1/2, CDK4 and PCNA nor for EMT/invadopodia markers such as MMP14, p-Vimentin (Ser56) and PARP, respectively. In contrast, the proliferation marker cyclin D1/2 was even reduced at higher concentrations in all 3 cell lines and CDK4 in the two breast cancer cell lines whereas PCNA levels stayed unaffected (Figure 1). Selected EMT/invadopodia markers remained generally unaffected upon treatment with BI 1002494. Only p-vimentin (Ser56) showed a decrease at 20 μM in DU4475. In contrast, the potent cyclin dependent kinase inhibitor p21 which binds to and inhibits the activity of cyclin-CDK2, -CDK1, and -CDK4/6 complexes, was dose-dependently upregulated in both breast cancer cell lines and to a lesser extend in MCF-10A (Figure 1). In order to analyse the influence of a possible spontaneous IgM production in the breast cell lines on SYK activation (for details see Discussion) we performed the assay in the presence and absence of anti-IgM f (ab)_2_ (Figure 1; +/– anti-IgM f (ab)_2_). Clearly, no difference (particularly no rescue effect) could be observed in the presence of anti-IgM F (ab)_2_ (Figure 1). Similar findings were observed with three structurally unrelated SYK inhibitors in MDA-MB-468 (Supplementary Figure 1A) and DU4475 cells (Supplementary Figure 1B), suggesting that this is an SYK-specific effect.

### Chronic incubation of MCF-10A, DU4475, MDA-MB-468 and T-47D cell lines with BI 1002494 does not increase cell number or increase proliferation and/or EMT/invadopodia markers

**Figure 2:**
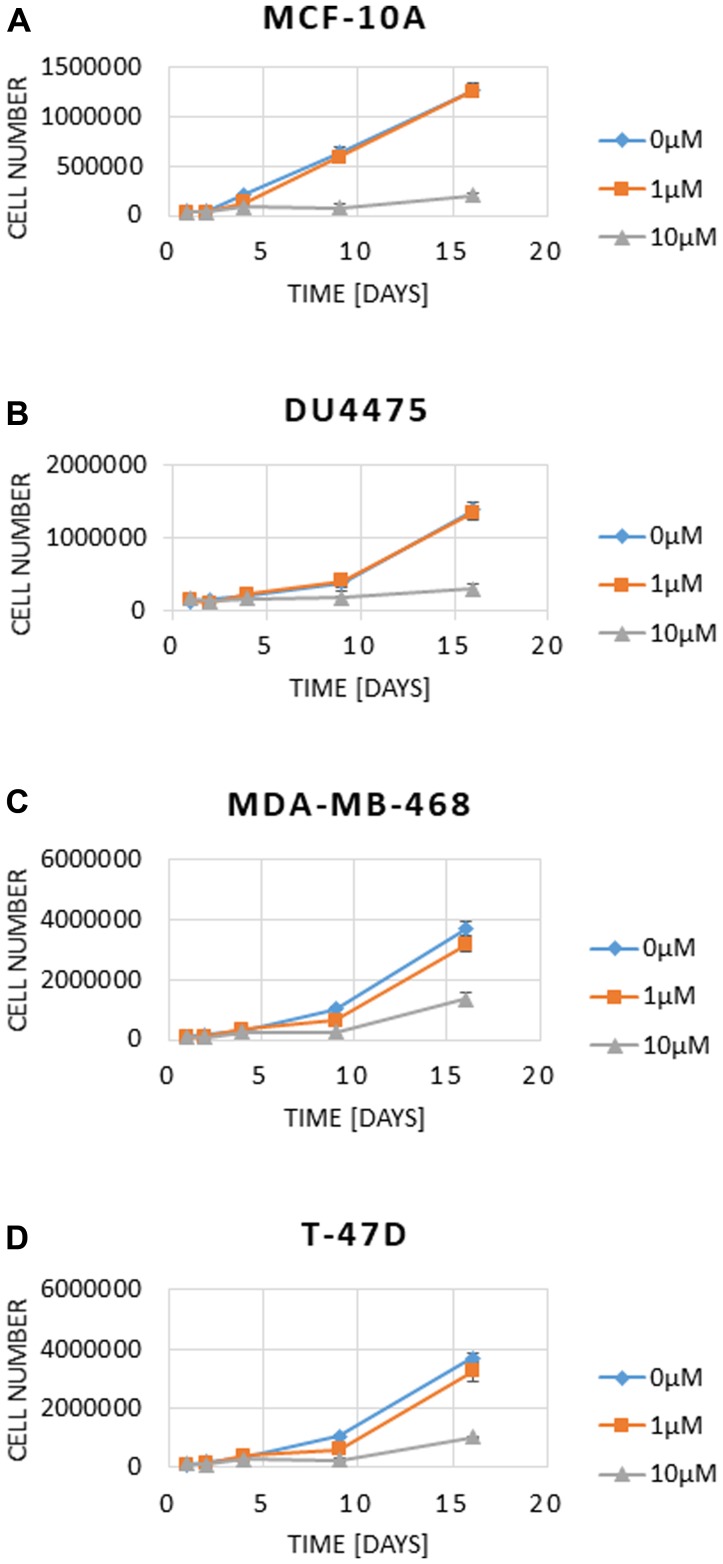
Effect of 16-day incubation of BI 1002494 (0, 1 or 10 μM) on cell number in MCF-10A (**A**), DU4475 (**B**), MDA-MB-468 (**C**) and T-47D (**D**) cell lines. Cell numbers and viability were analysed using a Vi-CellXR cell counter (Beckman Coulter) following the manufacturer’s instructions.

We next addressed the question if a long-term pharmacologic inhibition of SYK can change the proliferation rate *in vitro*. For this, cell lines MCF-10A (Figure 2A), DU4475 (Figure 2B) MDA-MB-468 (Figure 2C) and T-47D (Figure 2D) were incubated with 0, 1 and 10 μM BI 1002494 for 16 days and the cell number was determined at day 1, 2, 4, 8 and 16, respectively. Even after 16-day treatment with BI 1002494, there was no increase in cell number associated with 1 μM BI 1002494, but in contrast, 10 μM BI 1002494 was associated with a significant reduction in cell number of all 4 cell lines including the non-tumorigenic cell line MCF-10A. Next, we asked if a long-term pharmacologic inhibition of SYK in breast cancer cell lines could lead to an increase in proliferation and EMT/invadopodia markers.

**Figure 3:**
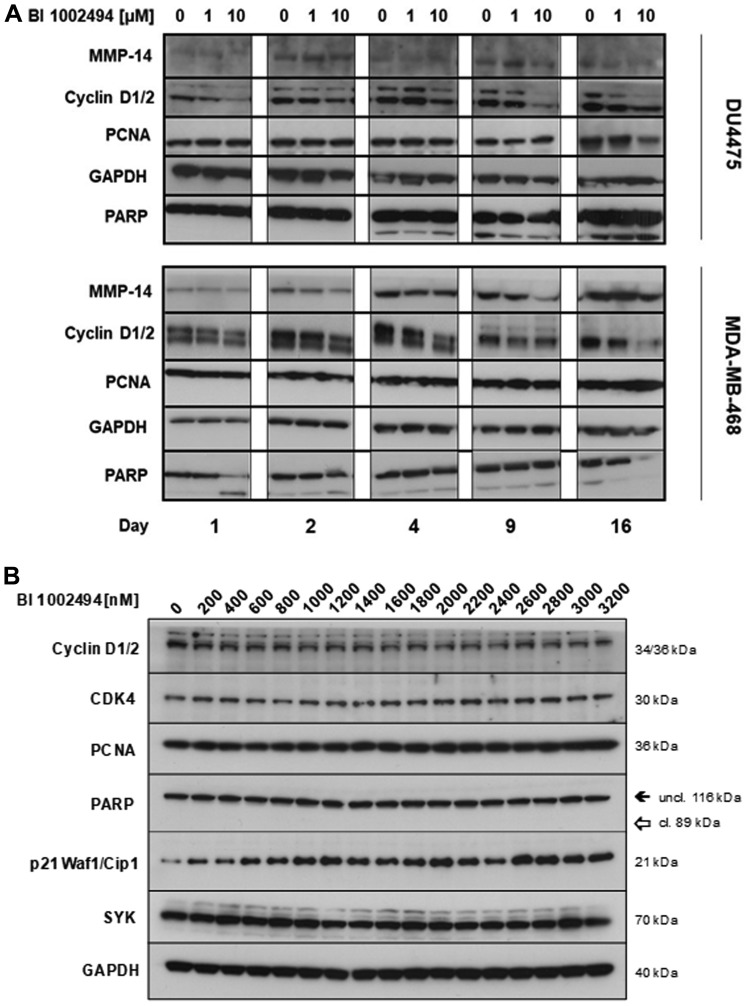
Effect of 16-day incubation of BI 1002494 (0, 1 or 10 μM) on selected proliferative (cyclin D1/2, PCNA) and EMT/invadopodia (MMP14, PARP) markers in DU4475 and MDA-MB-468 breast cancer cell lines (**A**) and effect of 13-day incubation of BI 1002494 at the indicated concentrations (0 to 3200 nM) on Cyclin D1/2, CDK4, PCNA, PARP, p21 (Waf1/Cip1) and SYK protein expression in the MDA-MB-468 breast cancer cell line (**B**).

Upon 16-day treatment with BI01002494 no specific increase in proliferation (cyclin D1/2, PCNA) or invadopodia (MMP14, PARP) markers is observed in either DU4475 or MDA-MB-468 (Figure 3A) breast cancer cell lines. In contrast, for some markers a reduction at higher concentrations and at longer incubation times (e. g. cyclin D1/2) was observed. Additionally, PARP cleavage was only seen at higher concentrations at an early time point (16 h) in MDA-MB-468 but not in the other two BC cell lines tested. However, long-term treatment 4–16 days (Figure 3A) leads also to slight induction of PARP cleavage in DU4475. In general, PARP cleavage seems not to be the main reason for inhibition of proliferation induced by BI 1002494 (see Figure 5). In order to analyse whether pharmacologic SYK inhibition goes hand in hand with its destabilization, MDA-MB-468 were incubated for 13 days with BI 1002494 in small increasing steps and SYK protein levels were measured and compared to some proliferation and invasion markers (Figure 3B). No change in SYK protein expression was observed as was seen for the other markers except for the anti-proliferative marker p21 that again exhibits a concentration-dependent increase.

### Incubation of MCF-10A and T-47D spheroids with BI 1002494 leads to reduction in tumour spheroid size

**Figure 4:**
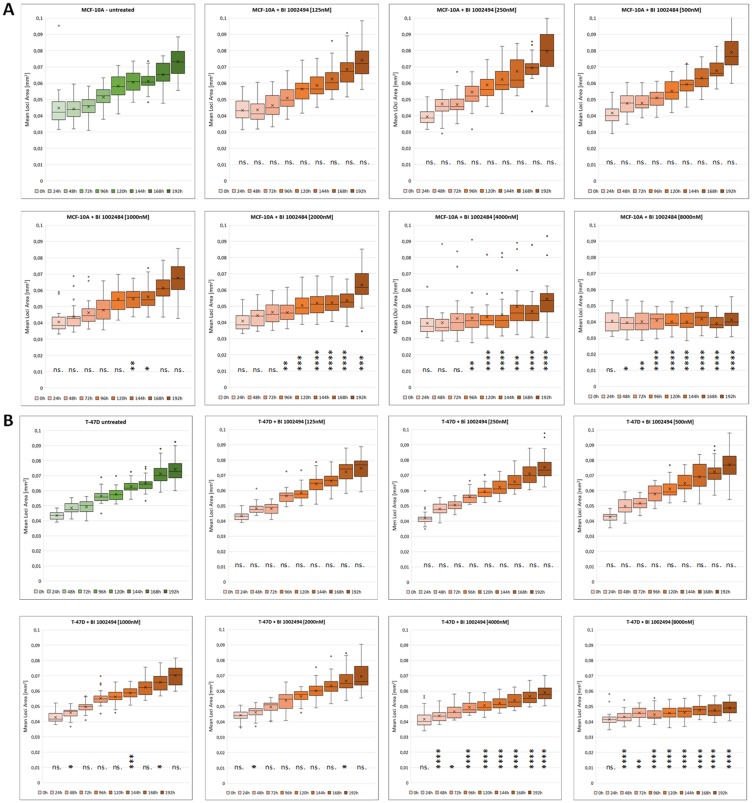
Box plot analysis of a time course experiment (0 h–192 h) with tumour spheroids of MCF-10A (**A**) and T-47D (**B**). 32 spheroids at each time point and for each concentration (0, 0.125, 0.25, 0.5, 1, 2, 4 and 8 μM) were non-invasively imaged on the Genetix CloneSelect Imager (CSI) which detected and recorded the area as the mean loci area [mm^2^] of spheroids. The line in the box shows the median, the asterisk indicates the mean value, the box shows the inter-quartile range (IQR), and the whiskers show the data within 1.5 × IQR of the upper and lower quartiles. Dots represent outliers. The mean loci area [mm^2^] of each spheroid at each time point and concentration (red box plots) was compared with the corresponding untreated sample (green box plots). Statistical analyses were performed using unpaired Student’s *t*-test. Significance levels are indicated as follows: ^*^ *p* < 0.05, ^**^ *p* < 0.01, ^***^ *p* < 0.001, ^****^ *p* < 0.0001; ns.: not significant.

To ask whether SYK inhibition has an effect on the 3-dimensional growth of an immortalized, non-carcinogenic epithelial breast cell line (MCF-10A) or on a carcinogenic epithelial cell (T-47D), spheroids were generated, seeded and BI 1002494 (0, 0.125, 0.25, 0.5, 1, 2, 4 and 8 μM) added 3 days after seeding. In general, no increases in spheroid size were observed (Supplementary Figure 2) or measured (Figure 4) compared to the corresponding untreated samples up to 8 μM BI 1002494 after 196 h (8 days) incubation. In contrast, a significant reduction in spheroid size was observed at higher doses (from 1 μM upwards) for both the non-transformed and carcinogenic breast cell line, MCF-10A (Figure 4A) and T-47D (Figure 4B), respectively. In general, MCF-10A exhibits a higher sensitivity towards treatment with BI 1002494 (Figure 4). Similar findings were observed in the T-47D spheroids with a structurally unrelated SYK inhibitor (data not shown), suggesting that this is an SYK-specific effect.

### SYK inhibition has no impact on the viability of human breast cancer cell line T-47D in organoid-like 3D cultures nor does it lead to a change in Ki67 levels

**Figure 5:**
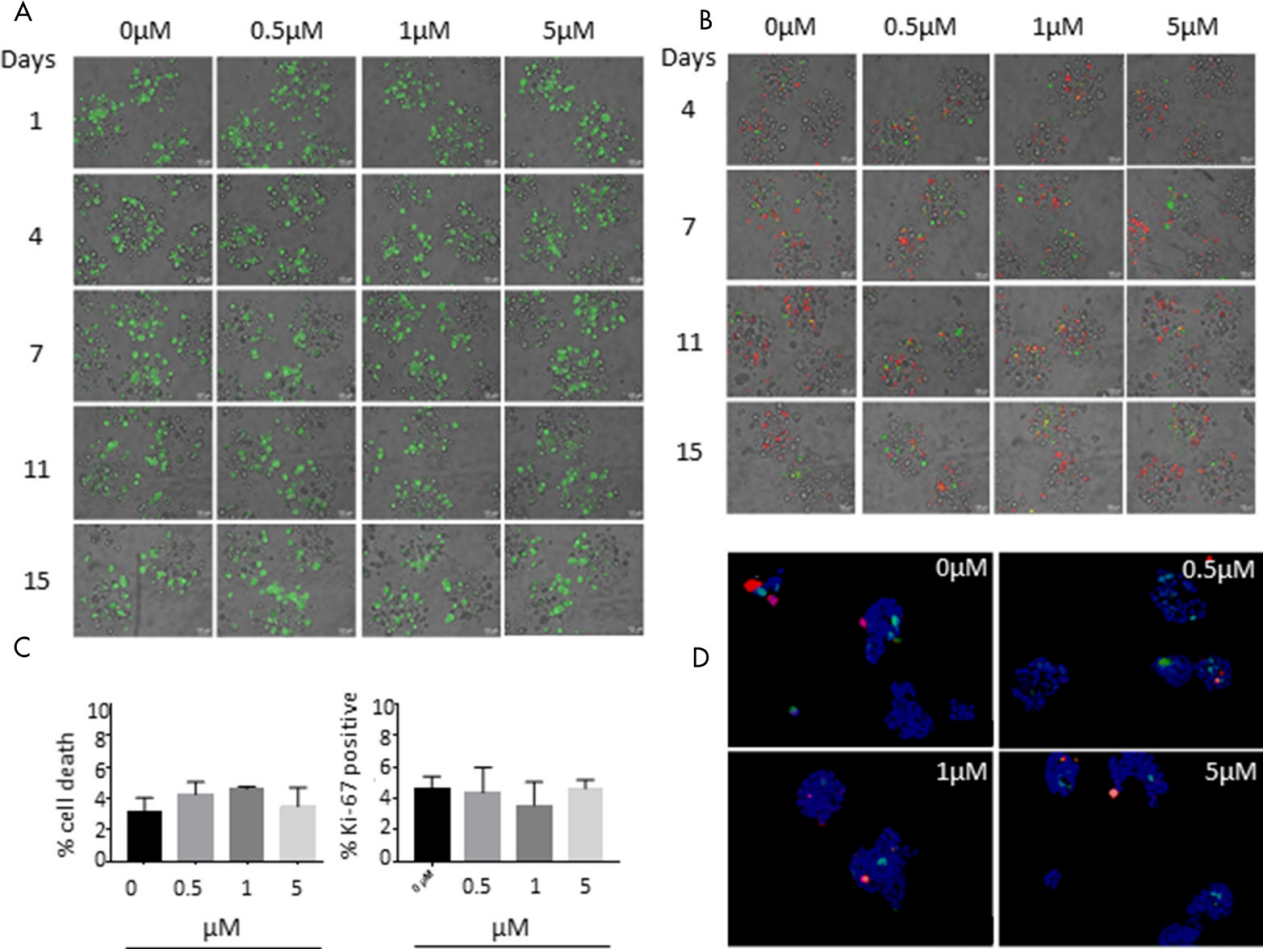
Effect of 15-day incubation of BI 1002494 on T-47D breast cancer cells cultivated in alginate capsules in a bioreactor. (**A**) Viability staining (FDA, fluorescein diacetate) and (**B**) Caspase (green) and annexin (red) live cell staining of 3D tumor cultures at different time points. (**C**) Cryosections of T-47D alginate capsules were stained for cell death (*In Situ* Cell Death Detection Kit, TMR red, Roche) and proliferation (Ki-67). Values are percent of stained positive cells compared to DAPI positive cells and are mean ± standard error of the mean (SEM) of three separate images. Statistical analysis was performed for each condition using Student’s *t* test and was non-significant (*P* > 0.5). (**D**) Cell death (*In Situ* Cell Death Detection Kit, TMR red, Roche) and Ki-67 (green) staining of 3D tumor cell cultures at day 15 after treatment.

In order to analyse the effect of BI 1002494 on the growth behaviour in a more complex 3D tissue culture setting, we applied an encapsulated bioreactor system that we have previously used to study immune cell infiltration into tumour spheroids and to characterize macrophage plasticity in the tumour microenvironment [23, 24]. For this, T-47D tumour spheroids were packed in alginate microcapsules and grown for one week in a stirred bioreactor followed by a two-week treatment with BI 1002494 (0.5, 1 and 5 μM) and DMSO (0.3%) as control (for technical details see Material and Methods). Viability staining (FDA, fluorescein diacetate; Figure 5A) and live cell staining of 3D tumour cultures (Caspase and Annexin; Figure 5B) at different time points revealed no significant differences between untreated and treated cultures. In addition, cryosections of T-47D alginate capsules were stained for cell death and proliferation (Ki-67) again showing no significant difference among the various experimental settings (Figure 5C and 5D).

### Effect of BI 1002494 on primary human mammary epithelial cells

**Figure 6:**
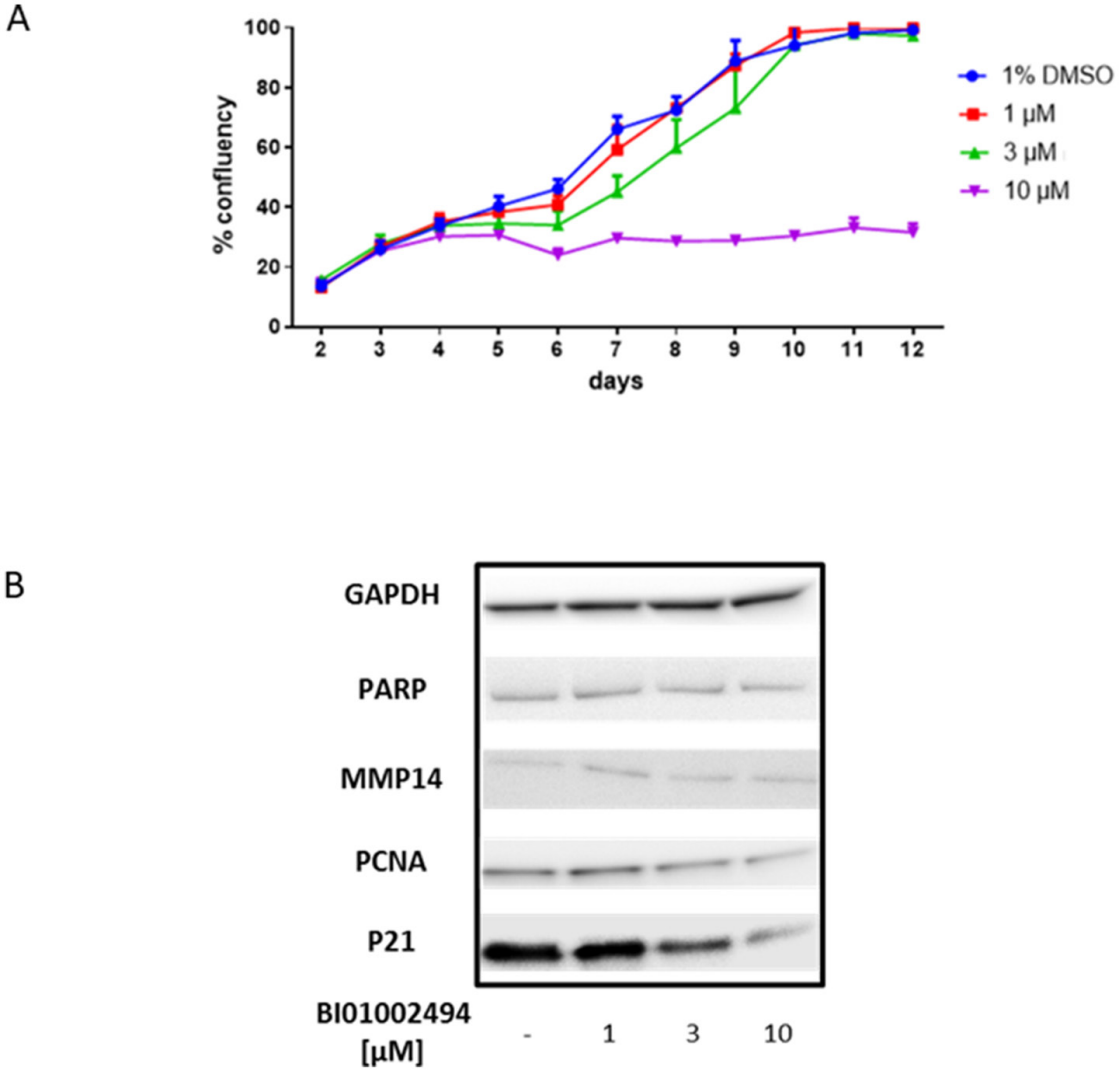
Effect of 12-day incubation of BI 1002494 (0, 1, 3, 10 μM) on primary human mammary epithelial cell proliferation (**A**) and 4-day incubation of BI 1002494 on PARP, MMP14, PCNA and p21 protein expression in primary human mammary epithelial cells (**B**).

To assess whether SYK inhibition had any effect on non-tumour breast epithelium, primary human mammary epithelial cells were incubated with BI 1002494 at 1, 3 or 10 μM for up to 12 days. Similar to the observations with the cancer cell lines, neither 1 or 3 μM of BI 1002494 showed any pro-proliferative effects, and again 10 μM was associated with a reduced cell number (Figure 6A). Due to lower protein recovery at the higher concentrations of BI 1002494 at the longer time points, the 4-day time point was selected for assessment of pro-proliferative and invadopodia markers. There was no observed change in protein levels of either PARP or MMP14 at any concentration of BI 1002494, and whilst lower concentrations of BI 1002494 did not alter protein levels of PCNA and p21, the highest concentration was associated with reduced levels of both PCNA and p21 (Figure 6B). In contrast to our data with tumour cell lines also the antiproliferative protein p21 was reduced, most likely because of toxic side effects and induction of cell death at this concentration (for details see Discussion).

### Effect of 13-week treatment with BI 1002494 in BALB/c mice

**Figure 7:**
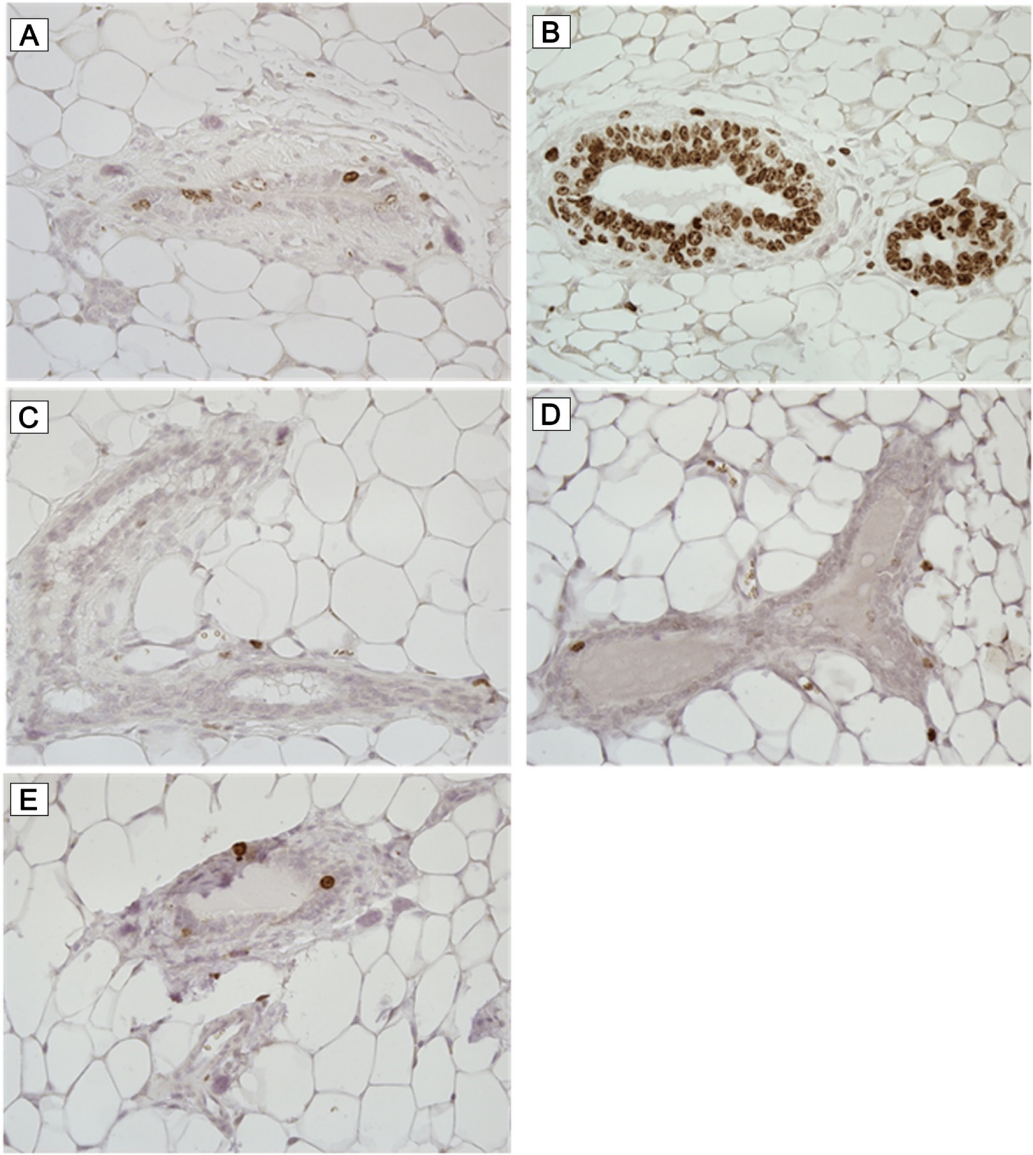
Ki-67 immunohistochemistry. Mice were treated daily for 13 weeks with either vehicle (**A** and **B**) or 30 mg/kg q. d. (**C**), 100 mg/kg q. d. (**D**) and 100 mg/kg b. i. d. (**E**) BI 1002494. Breast tissue was excised and stained for the proliferation marker Ki-67. Representative photomicrographs are illustrated.

Naïve adult mice were treated daily for 13 weeks with either 30 mg/kg qd, 100 mg/kg qd or 100 mg/kg bid BI 1002494. These doses provided IC_50_ coverage for 8, 16 and 24 hours respectively and the highest dose provided IC_90_ coverage for 16 hours (Supplementary Figure 3). The mammary gland excised and examined for ductal branching and cellular proliferation. No evidence of ductal branching was observed and qualitatively no increase in cells staining positively for the proliferation marker Ki-67 was observed (Figure 7). Quantification of the number of Ki-67 cells shows no increase in Ki-67 staining, broken down into low (< 20% cells), medium (20–60% cells) and high (> 60% cells) frequency (Table 1).

**Table 1:** Quantification of Ki-67 positive staining cells in ductal tissue from mice treated for 13 weeks with BI 1002494

Group	1	2	3	4
**Daily dose of BI 1002494 [mg/kg]**	**0**	**30**	**100**	**100 bid**
No. of specimens^*^ examined	18	18	20	16
Ki-67 positive nuclei^+^	12	12	12	15
Ki-67 positive nuclei^++^	3	5	5	1
Ki-67 positive nuclei^+++^	3	1	3	0

^*^one specimen per animal, ^+^low amount of Ki-67 positive nuclei (< 20% cells), ^++^slightly increased amount of Ki-67 positive nuclei (20% to 60% cells), ^+++^clearly increased amount of Ki-67 positive nuclei (> 60% cells).

### *In silico* analysis of SYK gene characteristics for tumour suppressor characteristics

Four breast cancer databases were assessed for focal deletion using single nucleotide polymorphism (SNP) Chip Profile in both SYK and PTEN genes (Table 2). Focal deletions in the SYK gene were not observed in any breast cancer sample, whereas focal deletions in the PTEN gene were observed in approximately 5% of the breast cancer samples. Furthermore, no statistically relevant lower expression levels of SYK were observed in a total of 30 infiltrating lobular and 269 infiltrating ductal breast carcinomas.

**Table 2:** Focal deletions in SYK and PTEN genes in breast cancer samples

**Database**	**SYK**	**PTEN**
Breast Clifford collection	0/159 (0%)	8/159 (5%)
Breast GEN-AU LCM samples	0/62 (0%)	3/62 (4.8%)
Breast Genentech collection	0/50 (0%)	1/50 (2%)
Tumorscape breast samples	0/193 (0%)	10/193 (5.2%)

The COSMIC database (Cancer Genome Project at the Sanger Institute; an online database of somatically acquired mutations found in human cancer) was assessed for mutations in both SYK and PTEN genes. No mutations were observed in the SYK gene in 201 breast cancer samples (0%) whereas mutations in the PTEN gene were observed in 60 samples from a total of 1097 breast cancer samples (5.5%). Overall, 5 mutations were observed in the SYK gene from a total of 1393 samples of all tumours, but no hot spot mutations were detected in the active site of SYK.

**Figure 8:**
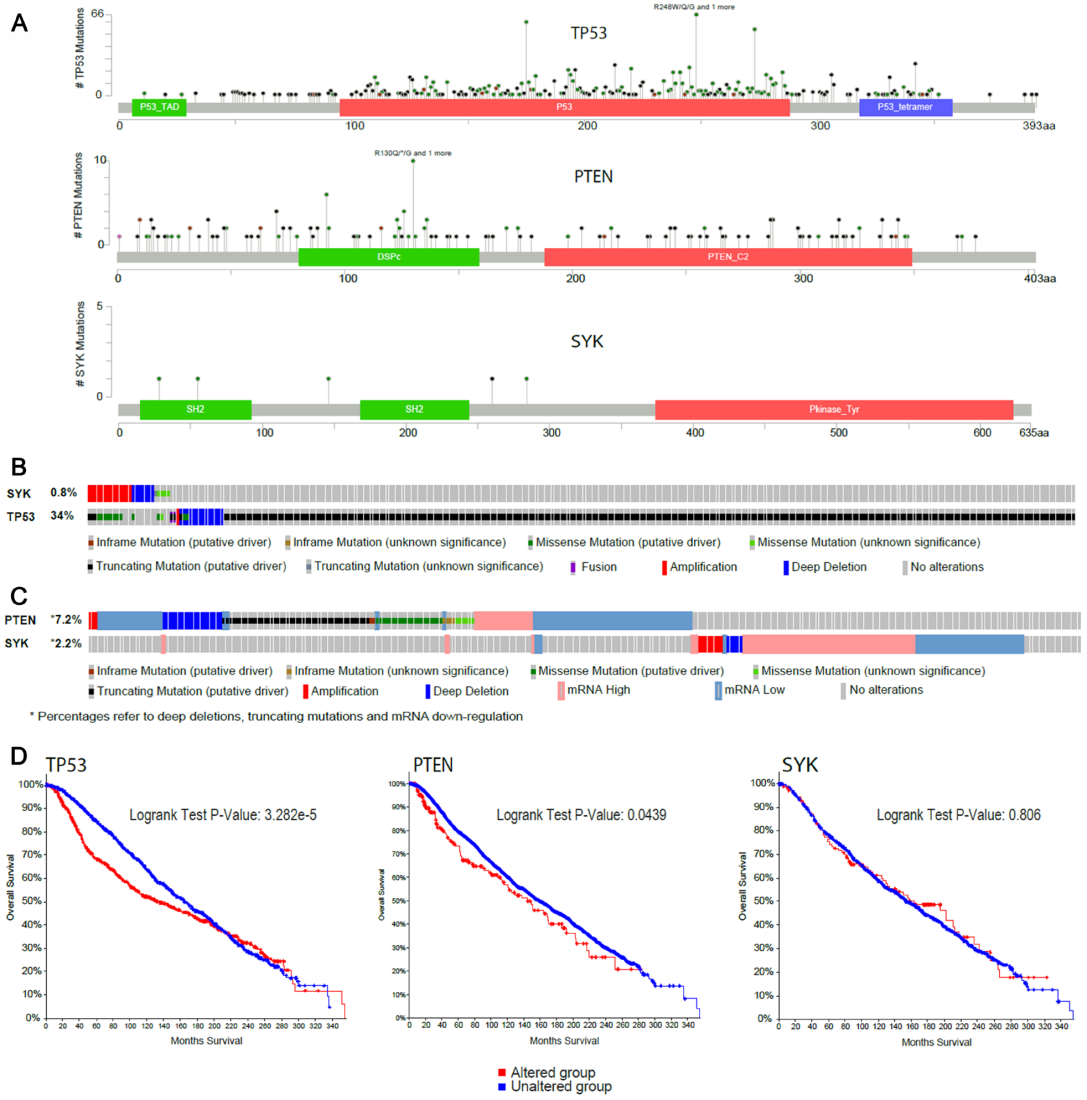
*In silico* analysis of SYK gene characteristics compared with the well-known tumour suppressors TP53 and PTEN in the Metabric and TCGA invasive breast cancer studies. (**A**) *Lollipop plot* of the number of mutations observed in SYK, TP53 and PTEN genes (TCGA and Metabric), (**B**) O*ncoprint* of the somatic DNA alterations rate in the SYK and TP53 genes (TCGA and Metabric), (**C**) *Oncoprint* of DNA mutations or mRNA expression alterations in SYK and PTEN genes (Metabric only) and (**D**) survival in patients with or without DNA alterations of known significance in TP53 and PTEN (TCGA and Metabric), as well as SYK (Metabric; mutations, copy number alterations and mRNA expression alterations).

The Cancer Genome Atlas (TCGA) database comprising 963 breast carcinoma samples mainly derived from invasive tumours (93.2%) and Metabric with a total of 2173 breast cancer samples (76.4% from invasive tumours) were used (via the online cBioPortal portal) following a molecular analysis to compare genetic characteristics of SYK and the well-known tumour suppressors TP53 and PTEN in samples from invasive breast cancer. SYK contained far fewer mutations compared to both PTEN and TP53. SYK was hardly mutated, with no nonsense mutations, no evidence that mutations lead to inactivation of SYK and no hot spot mutations in the kinase domain (Figure 8A). In comparison, TP53 was heavily mutated, containing many nonsense mutations (red dot), with almost all mutations reported to lead to inactivation of TP53 and hot spot mutations in both the P53 DNA binding domain and P53 tetramerization motif (Figure 8A and 8B). Similarly, PTEN was heavily mutated, with many nonsense and truncating mutations, as well as in-frame deletions, most of the mutations known to lead to inactivation of PTEN, and hot spot mutations were observed in the dual specificity phosphatase catalytic domain and the C2 domain of PTEN tumour-suppressor protein (Figure 8A and 8C). In contrast, the somatic mutation rate in the SYK gene was 0.8%, compared to 34% in the TP53 gene (Figure 8B). Furthermore, > 7% of breast cancer samples contained either deep depletions, truncating and in-frame mutations, missense mutations or mRNA downregulation in the PTEN, compared with 2.2% of samples containing mainly alterations in the mRNA levels (up- and downregulation), hardly amplification and deep deletions and no somatic mutations in the SYK gene (Figure 8C). Finally, in contrast to SYK, mutations in TP53 and PTEN lead to a significant reduction in overall survival (Figure 8D).

## DISCUSSION

A number of studies have suggested a negative regulatory role of SYK in the control of cell proliferation, migration and invasion in breast cancer cell lines [9, 10], that allelic deletion of SYK is associated with higher incidences of breast cancer [12], and that loss of SYK expression through promoter hypermethylation is associated with increased invasiveness of breast cancer cells [13]. However, it may be important for the therapeutic strategy for SYK inhibitors in oncologic and other indications to understand whether these putative effects are mediated through loss of the protein, which has been reported to have a number of protein-protein interaction functions (reviewed in [6, 25, 26]), and loss of the kinase activity.

SYK inhibition with BI 1002494, a potent and selective oral SYK kinase inhibitor [22] did not increase cell number or confluency in 2D culture or increase the spheroid size in 3D culture of any of these cell lines. Furthermore, neither in the spontaneously immortalized, non-tumorigeneic MCF-10A nor in the two breast cancer cell lines DU4475 and MDA-MB-468 was a detectable increase in any of the proliferation markers (CDK4, cyclin D1/2, PCNA) or EMT/invadopodia markers (MMP14, phospho-vimentin (Ser56) and PARP) observed (Figures 1 and 3). In contrast, levels of cyclin D1 and 2 are lower at higher concentrations of the SYK inhibitor. CDK4 and p21 are strongly affected mainly in the two cancer cell lines. PCNA, MMP14, phospho-vimentin (Ser56) and PARP cleavage stayed unaffected in all 3 cell lines. In contrast to pro-proliferative and invasive markers, an increase in the anti-proliferative protein p21 was observed (Figures 1 and 3).

These findings were also replicated in primary human mammary epithelial cells except for p21 where a decrease in p21 levels was observed (Figure 6B). Down-regulation of p21 has been described in primary cultures of human prostatic epithelial cells upon treatment with the natural product triptolide (PG490) [27]. As the p21 protein was shown in several cell types to antagonize p53-mediated apoptosis [28–30] down-regulation of p21 may therefore contribute to the ability to induce apoptosis. Nevertheless, treatment of primary human epithelial cells with BI 1002494 did not lead to an increase in proliferation (Figure 6A). These data suggest that inhibition of SYK kinase activity is not associated with increased cellular proliferation in either normal or malignant breast epithelial cells. This is supported by Wang *et al*. [31] who reported that the proto-oncogene supressing function of SYK is independent of SYK kinase activity (lack of transcription repressor activity with a kinase dead SYK) but is mediated via nuclear translocation of SYK and subsequent interaction with histone deacetylase. These data suggest and support our findings that kinase inhibition alone may not be associated with a pro-proliferative phenotype.

Furthermore, the SYK inhibitor, BAY61-3606, has been reported to reduce mammary epithelial cell (T-47D) acinar structure size in 3D culture, and this effect was synergistically enhanced in the presence of herceptin [32] which supports the findings reported here. Importantly, inhibition of SYK with BI 1002494 was not associated with loss of SYK protein, so at least in this test system it is possible to distinguish between SYK protein loss and inhibition of kinase activity. A decrease in cell number and spheroid size was observed at concentrations of BI 1002494 at 2 μM and higher, however, this may be a result of off-target inhibition of other kinases associated with cell cycle, such as PAK4 (75% inhibition at 1 μM) or AURA (66% inhibition at 1 μM) [22].

It has been shown that many human epithelial cancer cells - including breast cancer - also can express rearranged IgM transcript at a frequency of up to 50%. Moreover, CD79A and CD79B, were expressed as well on the cell surface of epithelial cancer cells and co-located with IgM leading to the formation of a functional B cell antigen receptor complex [33]. In these experiments, whilst CD79B does not show any expression in any of the 4 untreated cell lines, CD79A and CD22 exhibits a low expression in all 4 untreated cell lines. Moreover, during cultivation (dependent on the cell density) a low expression of all three genes was observed, indicating that these cells may be responsive to IgM stimulation. Most receptors coupled to the activation of SYK share in common a subunit or associated protein that contains at least one ITAM (for the BCR, these are CD79A and CD79B) [25, 33]. For instance, in B-cells the BCR-mediated activation of SYK can be achieved by addition of anti-IgM F (ab)_2_ thereby providing a polyclonal B-cell activating signal equivalent to a TI-2 antigen executed via the CD79-SYK-PI3K and/or CD79-SYK-PKC pathway [6, 20, 34]. Given the fact that in some epithelial cancer cells CD79A and CD79B co-located with IgM most likely forming the B cell antigen receptor complex leading to a survival signal in epithelial tumour cells [33], we therefore analysed the effect of SYK inhibition also in the presence of anti-IgM F (ab)_2_. Clearly, no difference in the sensitivity towards the SYK inhibitor (particularly no rescue effect) could be observed in the presence of anti-IgM F (ab)_2_ (Figure 1).

Additionally, there were no phenotype changes in organoid-like 3D cultures of either MCF-10A or T-47D cell lines (Figure 5). This is in apparent contrast to the findings of Coopman *et al.* [9] who showed that transfection of FLAG-tagged SYK back into an SYK-null invasive cell line (MDA-MB-435) reversed the invasive phenotype, which was reversed upon transfection of a kinase-dead SYK mutant. A second mutation with a functionally inactive SH2 domain also reversed this phenotype, but a third mutation in the auto-phosphorylated tyrosine residue in the SYK linker region (Y348F) maintained the non-invasive phenotype. The phosphorylation of Y348 (and Y352) enhances SYK signalling both by increasing the activity of SYK and by generating docking sites that mediate protein–protein interactions [25]. It is therefore unclear why in these experiments, Y348 phosphorylation does not contribute towards suppressing the invasive phenotype, whereas the kinase domain and SH2 domain both appear to contribute. One possible explanation is that the engineered mutations and/or incorporation of the FLAG-tag modify the protein scaffold such that either the nuclear translocation is compromised or the subsequent protein-protein interaction with histone deacetylase is blocked. This is perhaps further supported by the reported crystal structures of wild type and Y348 mutated SYK that suggests that the mutation in Y348 results in an auto-inhibited conformation of the kinase [35]. However, caution should be exercised with MDA-MB-435 as a model for breast cancer. The origin of the cell line, notwithstanding few contradictory reports, has been established to be of melanoma, reviewed in [36].

These *in vitro* observations were confirmed in naïve mice in which no histopathological evidence of ductal branching or cellular proliferation within the mammary gland was observed following 13 weeks treatment with BI 1002494.

Finally, an *in silico* genetic comparison of SYK with known tumour suppressor genes such as TP53 and PTEN across a number of breast cancer databases revealed no evidence that SYK has a typical tumour suppressor gene profile such as focal deletion, inactivating mutations or in-frame mutations. This is in contradiction to the findings of Blancato *et al.* [12] who reported that SYK deletion was highly prevalent in infiltrating breast cancers, although it should be noted that these conclusions were a result of the analysis of only 11 infiltrating breast cancer samples and 8 non-infiltrating breast cancer samples, and that SYK loss was observed in just 5 of the 11 infiltrating tumours and none of the 8 non-infiltrating tumours. In our analysis, we have collectively assessed data from over 3000 breast tumours available from public databases comprising 76.4% (1660/2173 in Metabric) and 93.2% (897/963 in TCGA) invasive breast cancer samples, respectively (Figure 8). In agreement with our findings, the very recent landmark analysis of somatic mutations detected in 560 human breast cancers did not find any mutations in the SYK gene [37], compared to highly significant mutation rates for the known proto-oncogenes TP53 and PTEN. Furthermore, in our own analysis of tumours from the Cancer Genome Atlas, mutations in TP53 and PTEN led to a significant reduction in overall survival in breast cancer patients, a finding not observed with mutations in SYK.

In conclusion, inhibition of kinase function of SYK does not apparently contribute to increased proliferation or invasiveness of breast cancer cell lines *in vitro* and there is no histopathological evidence in naïve mice for ductal branching or cellular proliferation within the mammary gland following 13 weeks treatment with BI 1002494. *In silico* analysis of genetic data from breast cancer databases reveals no evidence that SYK has a typical tumour suppressor profile.

## MATERIALS AND METHODS

### Reagents and test compounds

#### Primary antibodies

Cyclin D1/2 mouse monoclonal antibody (mAb) (1:1000; Sigma Aldrich #05-362), CDK4 mouse mouse mAb (1:2000; Cell Signaling Technology #2906), GAPDH mouse mAb (1:15.000; abcam #ab8245), Ki-67 mouse mAb (1:100; Dako, #M7240), MMP14 mouse mAb (1:1000; LSBio #LS-C8915), p21 Waf1/Cip1 rabbit mAb (1:1000; Cell Signaling Technology #2947), PCNA mouse mAb (1:2000; Cell Signaling Technology #2586), phospho-p53 (Ser15) mouse mAb (1:1000; Cell Signaling Technology #9286), phospho-vimentin (Ser56) rabbit polyclonal (1:1000; Cell Signaling Technology #3877), SYK mouse mAb (1:1000; abcam #ab3993), cleaved/uncleaved-PARP rabbit polyclonal (1:1000; Cell Signaling Technology #9542), anti-IgM F (ab)_2_ (Jackson Immuno Research Laboratories, #309-005-107).

#### Secondary antibodies

Anti mouse IgGs/HRP polyclonal goat (1:2000; Dako #P0447), anti rabbit IgGs/HRP polyclonal goat (1:2000; Dako #P0448).

The dilution ratio and provider for each antibody are indicted in brackets. Blocking solution for all antibodies was a 5% dry milk solution except for anti-SYK antibody (10% BSA).

BI 1002494 was dissolved in DMSO (Sigma-Aldrich, #41648) as a 10 mM stock solution and serial dilutions prepared in DMSO prior to final dilution in assay buffer. Final DMSO concentration was 0.3%.

### Cell lines and cell culture

Primary human mammary epithelial cells (Lonza, #CC-2551) were cultured in MEGM Mammary Epithelial Cell Growth Medium (Lonza, #CC-3150). Human MCF-10A mammary epithelial cells (ATCC, #CRL-10317) were cultured in MEBM medium (Lonza, #CC-3151) supplemented with bovine pituitary extract, human epidermal growth factor, hydrocortisone and insulin (Lonza, #CC-4136 MEGM SingleQuot Kit) and 100ng/mL cholera toxin (Sigma-Aldrich, #C8052). Human breast carcinoma DU4475 (ATCC, #HTB-123) and MDA-MB-468 (ATCC, #HTB-132) cell lines were cultured in RPMI medium with 10% fetal calf serum (FCS) and penicillin-streptomycin (all from Invitrogen). Human ductal breast T-47D (ATCC, #HTB-133) epithelial tumour cells were cultured in IMDM medium (Lonza, #12-722F) supplemented with 10% FCS and penicillin-streptomycin (all from Invitrogen). For cultivation in stirred encapsulated bioreactor cultures, T-47D was grown in a phenol red free RPMI medium (for details see Encapsulated Stirred Bioreactor Culture). Cell numbers and viability were analysed using a Vi-CellXR cell counter (Beckman Coulter) following the manufacturer’s instructions.

### Spheroid formation

Spheroids were cultured in medium supplemented with medium-specific methylcellulose. Methylcellulose solution (Sigma-Aldrich, #M0512 was made by adding 1.6 g of autoclaved pure methylcellulose powder to 50 mL of 60°C basal medium in a 100 mL conical flask and stirring magnetically for 10 minutes. 50 mL cold basal medium was then added and stirred overnight at 4°C. The solution was aliquoted into two 50 mL falcon tubes and cleared by centrifugation at 4600 rpm for 2 hours at 4°C. The clear supernatant was aliquoted further into 50mL falcon tubes at 10 mL each and stored at –20°C.

MCF-10A and T-47D cells were seeded in U-bottomed, 96-well suspension culture plates (PerkinElmer, #ISP-06-001) at 500 cells per well. Cells were collected by centrifugation and re-suspended in 100 μL medium containing 5% FCS and methylcellulose in a 4:1 dilution. They were then left in an incubator at 37°C and 5% CO_2_ for 3 days to form spheroids.

### Proliferation assays

24-well plates were seeded at 10 000 cells per well in 500 μL medium. They were imaged daily on the CSI (CloneSelect Imager, Molecular Devices) which measured the confluence (representing cell number) of the wells, to generate a growth curve. 200 μL medium was added to each well every three days. Plates were incubated at 37°C, 5% CO_2_ in a loosely sealed box containing damp paper towels to reduce the effects of evaporation in the outside wells.

In a 96-well plate cells were seeded at a density of 5000 cells in 120 μL media per well. After 72 hours, Alamar blue reagent (Invitrogen) was added to the wells in a 1:10 dilution. Fluorescence was then measured every hour for 3 hours using a SPECTRAmax, GEMINI XPS fluorescence reader (Molecular Devices).

### Spheroid proliferation assays

Once the spheroids had formed, they were analysed using the CellTiter-Glo Luminescence cell viability assay (Promega, #G7570). Spheroids were transferred to opaque, flat-bottomed 96-well plates (PerkinElmer, white #6005290 or black #6005270). CellTiter-Glo substrate was added to CellTiter-Glo buffer to make the CellTiter-Glo reagent that was then added to the wells at a 1:1 dilution and the plate was placed on a shaker for 2 minutes. Luminescence was then measured on an EnVision 2101 Multi Label Recorder (Perkin Elmer).

### Encapsulated stirred bioreactor culture

After aggregation of tumour cell line T-47D, tumour spheroids were encapsulated essentially as described [19]. For encapsulation GRGDSP peptide-coupled MVG alginate from NovaMatrix was used (NovaMatrix, #4270129). Capsules containing T-47D tumour spheroids were cultivated in a DASbox^®^ Mini Bioreactor System (Eppendorf, #76DX04CCSU) utilizing BioBLU^®^ 0.3 Single-Use Vessels (Eppendorf) essentially as described [19]. The culture medium was RPMI (without phenol red) supplemented with 10% FCS and penicillin-streptomycin (all from Invitrogen). For long term treatment over 15 days, after one week 50 mL medium was taken out of the vessel and replaced by 50 mL fresh medium containing the compound with the respective concentration (0.5, 1 and 5 μM BI 1002494 and DMSO as control (0.3%), respectively). Samples were taken each 3–4 days to stain for viability (Fluorescin diacetate, ThermoFisher Scientific, #F1303) and apoptosis (NucView, 488 Caspase-3 Enzyme Substrate, Biotium, #10402).

### Western blot analysis

Cells were lysed using lysis buffer containing 20 mM Hepes pH 7.4, 100 mM NaCl, 5 mM EDTA pH 7.4, 1 mM Na_3_VO_4_, 30 mM NaF, 5% Glycerol, 0.1% SDS, 1% Triton X-100, 10 mM p-Nitrophenylphosphate and 1 mM β-Glycerophosphate. Just before lysis, 5 mM DTT, 0.002% PMSF, 0.5% Phosphatase inhibitor cocktail 1 (Sigma-Aldrich, #P2850) and 0.5% phosphatase inhibitor cocktail 2 (Sigma-Aldrich, #P5726), and a Complete Mini EDTA-free protease inhibitor cocktail tablet (Roche, #11836170001) were added. 120 μL of buffer was added to each well of a six well plate and left on ice for 5 minutes.

A Bradford test was carried out to ascertain protein concentration according to manufacturer’s guidelines (Bio-Rad). For a XT Precast 26-well polyacrylamide gel (Fermentas), 15 μg of protein was loaded with 4× Roti Load buffer (Roth) and water to a volume of 15 μL as well as 5 μL of Full-Range rainbow molecular weight marker (GE Healthcare, #RPN756E). For the XT Precast 18-well polyacrylamide gel (Fermentas), 30 μg of protein per lane was loaded with 4× Roti-Load buffer and water to a volume of 30 μL, along with 9 μL of the rainbow marker. The gels were run at 130V for approximately 1.5 hours, or until the samples reached the bottom of the gel.

The gel was transferred to a Bio-Rad PVDF blotting sandwich (the membrane was deactivated in methanol first), soaked in Towbin buffer (70% ddH_2_O, 20% methanol and 10% Tris/Glycine buffer (Bio-Rad, #1610734) for 5 minutes and placed in a TE77XP Semi-Dry Blotter (Hoefer) for 1.75 hours at 70 mA per gel. Once transferred, the membrane was blocked in 5% milk TBS-T for 1 hour before the primary antibodies were added (see antibodies section) and incubated overnight at 4°C on a shaker. The membranes were then washed 4 times for 10 minutes in TBS-T before the secondary antibody was added with 5% milk TBS-T and incubated for 1 hour at room temperature on a shaker. After 4 further 10 minute washes, the membrane was exposed to enhanced chemo-luminescence (ECL) Western blotting reagents (Amersham) for 30 seconds on each side and detected using high performance autoradiography film (Amersham).

### Mouse study

Adult, test naïve, female Balb/c mice (8–10 weeks; approx. 18–23 g) were purchased from Charles River (Charles River Laboratories International Inc., Sulzfeld, Germany). Animals were kept in rooms maintained at constant temperature (22°C ± 2°C) and humidity (60% ± 15%) under a 12-hour light-dark cycle. The animals were housed in isolated ventilated cages and allowed free access to water and standard food. All animal experimentation was conducted in accordance with German national guidelines and legal regulations and approved by the ethical committee Regierungspräsidium Tübingen (Germany) and with the ARRIVE guidelines.

Animals were treated daily with either vehicle or BI 1002494 (either 30 mg/kg q. d., 100 mg/kg q. d. or 100 mg/kg b. i. d.) dissolved in 0.5% hydroxyethylcellulose containing 0.01% Tween-20 and applied by oral gavage.

After 13 weeks of treatment, animals were euthanised with an overdose of pentobarbital. The mammary gland was excised and immersed immediately into fixative (4% buffered formaldehyde) in order to minimize any *ex vivo* artefacts. Samples were then dehydrated and embedded in paraffin and from each of the FFPE tissue blocks, sequential sections (4 μm in thickness) were generated in a paraffin-cutting microtome.

The IHC procedure was automated using an automated immunohistochemistry robot (Autostainer Plus, Dako). Briefly, sections were pre-treated with serum free protein block (Agilent Dako, #X090930-2) for 10 minutes before the incubation with the primary anti-Ki-67 mouse monoclonal antibody (Dako, #M7240). After a washing step the primary antibodies were detected by secondary antibodies conjugated to HR-Peroxidase (HRP) and DAB (brown chromogen). Hematoxylin was used as background staining (blue nuclei), the tissue was dehydrated, and the slides were then mounted under glass coverslips with Pertex mounting medium (Leica Microsystems, #LEIC811).

### *In silico* genetic evaluation of SYK for tumour suppressor characteristics

*In silico* analysis was performed using these publically available databases:

Breast Clifford collection, Breast GENAU LCM samples, Breast Genentech collection, Tumorscape breast samples, COSMIC database. The Cancer Genome Atlas (TGCA) [11].

SYK gene characteristics were compared with the well-known tumour suppressors TP53 and PTEN for the number of mutations, somatic mutation rate, number of deep depletions, truncating mutations or mRNA downregulation in patient samples. Furthermore, the number of disease-free periods and survival in patients with or without alterations in SYK was compared to those with alterations in TP53 and PTEN genes.

## SUPPLEMENTARY MATERIALS


