# Supplementary material for: Inhibition of SYK kinase does not confer a pro-proliferative or pro-invasive phenotype in breast epithelium or breast cancer cells
Source: Oncotarget. 2020 Apr 7;11(14):1257–72. doi: 10.18632/oncotarget.27545 (PMC7147091; doi:10.18632/oncotarget.27545)
Supplement: Supplementary file 1 [file oncotarget-11-1257-s001.pdf]

Inhibition of SYK kinase does not confer a pro-proliferative or pro-invasive phenotype in breast epithelium or breast cancer cells

SUPPLEMENTARY MATERIALS

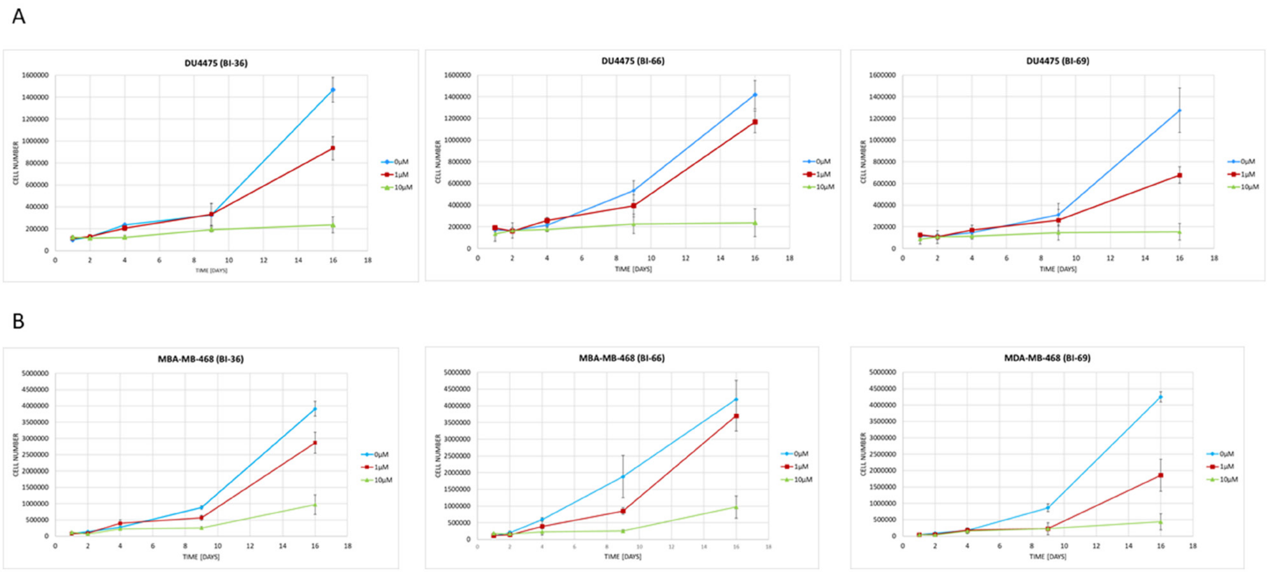

Supplementary Figure 1: Effect of 16-day incubation with 3 structurally distinct SYK inhibitors, BI-36, BI-66 and BI-99 (0, 0.125, 0.25, 0.5, 1, 2, 4 and 8 µM) on cell number in (**A**), DU4475 (**B**) and MDA-MB-468 cell lines. Cell numbers and viability were analysed using a Vi-CellXR cell counter (Beckman Coulter) following the manufacturer's instructions.

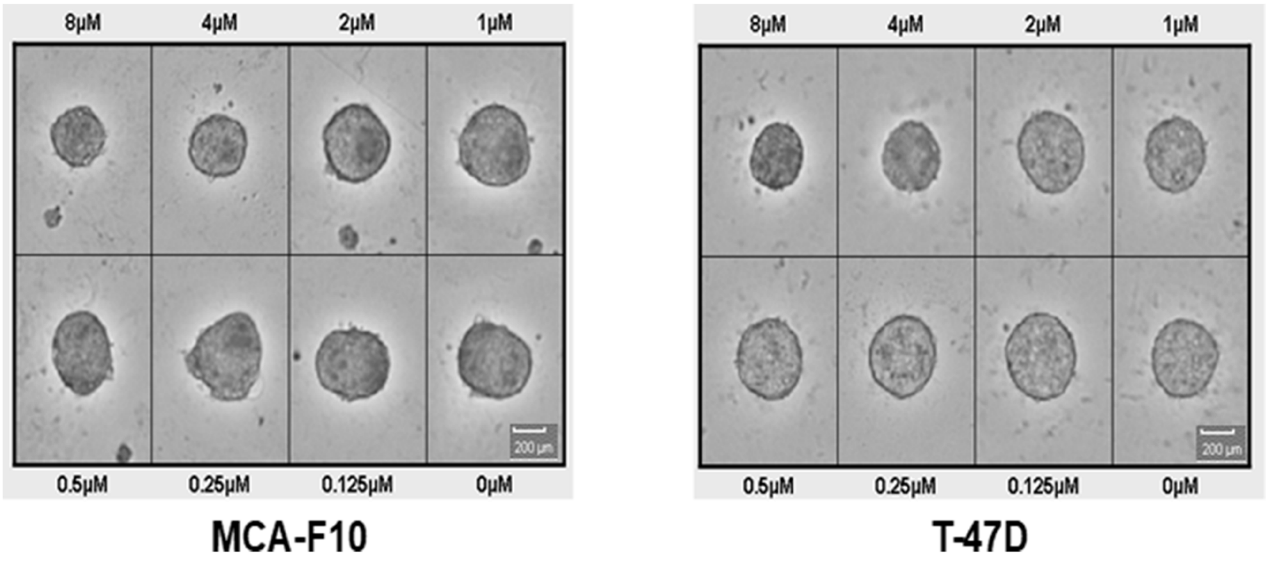

Supplementary Figure 2: Effect of 6-day incubation of BI 1002494 (0, 0.125, 0.25, 0.5, 1, 2, 4 and 8 μM) on spheroids derived from MCA-F10 (**left panel**) and T-47D (**right panel**). U-bottomed plates containing the spheroids were non-invasively imaged on the Genetix CloneSelect Imager (CSI) which detected and recorded the area of the spheroids as an indication of growth. Representative pictures taken after a 144 h incubation with BI 1002494.

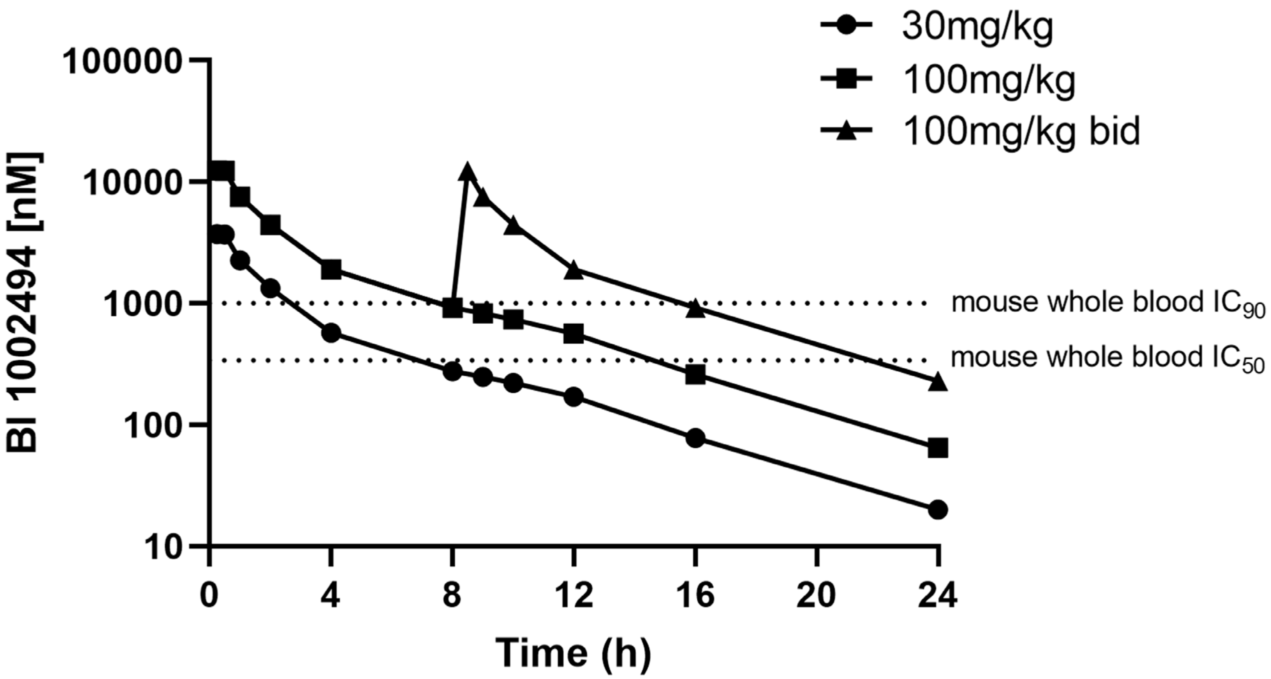

Supplementary Figure 3: Simulated pharmacokinetic profile of BI 1002494 dosed orally at 30 mg/kg and 100 mg/kg once daily and 100 mg/kg twice daily. Data is extrapolated from a one daily 10 mg/kg PK profile based on the known linear dose-exposure relationship for BI 1002494.
